# 4-1BB Signaling Activates the T Cell Factor 1 Effector/β-Catenin Pathway with Delayed Kinetics via ERK Signaling and Delayed PI3K/AKT Activation to Promote the Proliferation of CD8^+^ T Cells

**DOI:** 10.1371/journal.pone.0069677

**Published:** 2013-07-11

**Authors:** Do Y. Lee, Beom K. Choi, Don G. Lee, Young H. Kim, Chang H. Kim, Seung J. Lee, Byoung S. Kwon

**Affiliations:** 1 Cancer Immunology Branch, Division of Cancer Biology, National Cancer Center, Ilsan, Goyang, Gyeonggi-do, Korea; 2 Immune Cell Production Unit, Program for Immunotherapeutic Research, National Cancer Center, Ilsan, Goyang, Gyeonggi-do, Korea; 3 Section of Clinical Immunology, Allergy, and Rheumatology, Department of Medicine, Tulane University Health Sciences Center, New Orleans, Louisiana, United States of America; National Cancer Institute (INCA), Brazil

## Abstract

4-1BB (CD137), an inducible costimulatory molecule, strongly enhances the proliferation and effector function of CD8^+^ T cells. Since the serine/threonine kinase, glycogen synthase kinase-3 (GSK-3), is involved in a variety of signaling pathways of cellular proliferation, migration, immune responses, and apoptosis, we examined whether 4-1BB signaling activates GSK-3/β-catenin signaling and downstream transcription factors to enhance the proliferation of CD8^+^ T cells. 4-1BB signaling induces rapid activation of ERK and IκB degradation, and shows delayed activation of AKT at 24 h post 4-1BB stimulation on anti-CD3 activated T cells. ERK and AKT signals were required for sustained β-catenin levels by inactivating GSK-3, which was also observed with delayed kinetics after 4-1BB stimulation. As a transcriptional partner of β-catenin, 4-1BB signaling decreased levels of FOXO1 and increased levels of stimulatory TCF1 in CD8^+^ T cells at 2–3 days but not at early time points after 4-1BB engagement. The enhanced proliferation of CD8^+^ T cells due to 4-1BB signaling was completely abolished by treatment with the TCF1/β-catenin inhibitor quercetin. These results show that 4-1BB signaling enhances the proliferation of activated CD8^+^ T cells by activating the TCF1/β-catenin axis via the PI3K/AKT/ERK pathway. As effects of 4-1BB on AKT, FOXO1, β-catenin and GSK-3β showed delayed kinetics it is likely that an intervening molecule induced by 4-1BB and ERK signaling in activated T cells is responsible for these effects. These effects were observed on CD8^+^ but not on CD4^+^ T cells. Moreover, 4-1BB appeared to be unique among several TNFRs tested in inducing increase in stimulatory over inhibitory TCF-1.

## Introduction

The T cell costimulatory receptor 4-1BB (CD137) is induced on activated T cells and plays a variety of crucial roles: preventing activation-induced cell death (AICD), promoting cell cycle progression, enhancing cytotoxicity and the production of type 1 cytokines such as IL-2, IFN-γ, and TNF-α, and increasing the memory CD8^+^ T cells [1], [2]. Previous studies have demonstrated that 4-1BB signaling triggers TRAF-dependent NF-κB activation to increase the expression of anti-apoptotic proteins including Bcl-2 and Bcl-X_L_, and activates the PI3K and MEK-1/2 signaling pathway to promote cell cycle progression [3], [4].

*In vivo* 4-1BB triggering with agonistic antibodies enhances CD8^+^ T cell responses against tumors, and provides adjuvant-like functions in combination with various types of anti-cancer therapeutics [5]. 4-1BB/4-1BBL interactions are also considered positive regulators of CD8^+^ T cell responses against viruses such as influenza virus, lymphocytic choriomeningitis virus (LCMV), and herpes simplex virus (HSV) [6]–[8]. The effect of 4-1BB/4-1BBL interactions, however, may be both positive and negative in viral infections depending on the type of virus and timing of mAb administration [7], [9]–[11]. 4-1BB signals *in vivo* can be further modulated in CD8^+^ T cells by other pathogen-induced factors.

CD8^+^ T cells require signals for survival, cell cycle progression, biomass formation, and differentiation into effector and memory cells. 4-1BB has been known to use TRAF1/2, PI3K, IKK, and mitogen signaling pathways to enhance CD8^+^ T cell responses [12]. Although it is well known that 4-1BB uses NF-κB for cytokine induction and survival of CD8^+^ T cells, other transcription factors that mediate the effects of 4-1BB are poorly understood.

Glycogen synthase kinase-3 (GSK-3) is involved in a variety of signaling pathways of cellular proliferation, migration, inflammation and immune responses, glucose regulation, and apoptosis [13]. GSK-3 is not only necessary for the inflammation induced by innate immune cells [14], but also required to modulate proliferation, survival, differentiation and anergy of T cells [15]. In particular, the inactivation of GSK-3β by phosphorylation of the regulatory serine residue at position 9 is critical to preventing AICD of CD4^+^ and CD8^+^ T cells [16] and over-expression of constitutively active GSK-3β decreases proliferation of CD8^+^ T cells [17]. GSK-3β activation increases β-catenin level and interaction of β-catenin with T cell factor 1 (TCF1) family transcription factors regulate the proliferation and differentiation of CD8^+^ T cells [18]. Therefore, we examined whether 4-1BB signaling would modulate GSK-3β-mediated signaling pathway to enhance the CD8^+^ T cell responses. Here we provide the evidence that 4-1BB signaling activates the β-catenin/TCF1 pathway with delayed kinetics through rapid ERK signaling and delayed PI3K/AKT activation to enhance CD8^+^ T cell responses.

## Materials and Methods

### Mice, reagents, and antibodies

All animal studies were approved by the Institutional Animal Care and Use Committee (IACUC) review board of National Cancer Center (NCC-10-080) and conducted under the guidelines of the National Cancer Center IACUC. Six-to-eight-week-old C57BL/6 mice were purchased from OrientBio (Gapyoung, Korea). 4-1BB-deficient (4-1BB^−/−^) C57BL/6 mice were generated as previously reported [19]. Anti-CD3 mAb (clone 145-2C11) and biotin- and PE-labeled anti-CD8β mAb were purchased from BD Pharmingen (San Diego, CA), and CD4− and CD8−microbeads from Miltenyi Biotech (Auburn, CA). Agonistic anti-4-1BB mAb (3E1) was a kind gift from Dr. Robert Mittler, (Emory University, Atlanta, GA), anti-GITR mAb (DTA-1) from Dr. Simon Sakaguchi (Kyoto University, Kyoto, Japan), and anti-OX40 mAb (OX86) from Dr. Michael Croft (La Jolla Institute, CA). Anti-CD27, anti-CD30, anti-CD4-FITC, anti-CD8-FITC, and anti-4-1BB-PE mAbs were from eBioscience (San Diego, CA). CTLA-4Ig was from AdipoGen, Inc. (Incheon, Korea). LY294002 and PD98059 were from Calbiochem (San Diego, CA). Quercetin was purchased from Acros (Belgium, Geel). All antibodies for Western blotting including anti-AKT, anti-phospho-AKT, anti-phospho-GSK-3β, anti-GSK-3β, anti-phospho-ERK, anti-ERK, anti-IκB, anti-TCF1, and anti-FOXO1 were purchased from Cell Signaling (Danvers, MA) except anti-β-catenin mAb (Santa Cruz Biotechnology, CA).

### Purification of CD8^+^ and CD4^+^ T cells

Lymphocytes from the spleens and lymph nodes (LNs) of wild type C57BL/6 mice and 4-1BB^−/−^ mice were resuspended in PBS containing 5% FBS at concentrations of 10^8^ cells/ml, incubated with CD4− or CD8−microbeads at 4°C for 15 min and loaded on LS columns to isolate CD4^+^ T or CD8^+^ T cells. The purified CD4^+^ T and CD8^+^ T cells were routinely >95% pure by flow cytometry.

### T cell stimulation

Purified CD8^+^ T cells were plated at 5×10^5^ cells/well in 96-well round-bottom microplates with 0.1 µg/ml of anti-CD3 (BD Pharmingen). After 16 h, samples were stained with anti-4-1BB-FITC and anti-CD8-PE, and the percentage of 4-1BB^+^CD8^+^ T cells was routinely ∼70% by flow cytometry. After the 4-1BB^+^CD8^+^ T cells were counted, they were further stimulated with 5 or 10 µg/ml of the agonistic form of antibodies against TNFRSF members. To block the activity of PI3K or ERK, the activated CD8^+^ T cells were first pre-incubated with the appropriate pharmacological inhibitors for 1 h and then stimulated with anti-4-1BB. Purified CD4^+^ T cells were plated in the same manner as the CD8^+^ T cells and incubated with 0.1 µg/ml anti-CD3 for 36 h, which yielded ∼50% of 4-1BB^+^CD4^+^ T cells by flow cytometry, and were further treated with anti-4-1BB in the presence or absence of pharmacological inhibitors.

### CFSE labeling and IFN-γ assay

Purified T cells were resuspended in 1×PBS at 1×10^7^ cells/ml and stained with 1 µM CFSE (1 mM CFSE in DMSO) for 5 min at 37°C. Next, the cells were incubated with ice-cold FBS for 1 min, washed three times with complete RPMI medium, and resuspended in complete RPMI medium. CFSE-labeled T cells were plated at 3×10^5^ cells/well in 96-well round-bottom microplates and stimulated with 0.1 µg/ml of anti-CD3 for 16 h followed by anti-4-1BB or rat IgG for another 48 h. The dilution of CFSE used was determined by flow cytometry. IFN-γ concentration in cell supernatants was determined using “Th1/Th2 mouse cytokine CBA” kit (BD Bioscience) and assays were carried out following the manufacturer's instructions.

### Western blotting

CD4^+^ T and CD8^+^ T cells were lysed with a gentle lysis buffer (10 mM Tris pH 7.5, 5 mM EDTA, 150 mM NaCl, 1% Triton X-100, 1 mM PMSF, and protease inhibitors) and extracts were prepared by removing cell debris by centrifugation. Extracted proteins were separated on 12% SDS-PAGE gels and transferred to polyvinylidene fluoride (PVDF) membranes. Proteins of interest were detected with primary Abs and secondary HRP-labeled Ab, and bound Ab was detected by ECL (Intron, Seoul, Korea).

### RT-PCR

Total RNAs were extracted from CD8^+^ T cells with Trizol reagent (Invitrogen), and first-strand cDNA was synthesized with 0.5 µg of total RNA using a reverse transcription system kit (Promega, Madison, WI) and oligo(dT)_12–18_. PCR was performed with the cDNAs and specific primer sets. The PCR primers used were as follows: *Wnt3a*, forward 5′-CGGAAGGAGGAGTTCCTGAG-3′, reverse 5′-ACTCTGGGGGACTG CAAATC-3′; *Wnt5a*, forward 5′-CTGTCTTTGGCAGGGTGATG-3′, reverse 5′-TAGCGTC CACGAACTCCTTG-3′; *Wnt10b*, forward 5′-TTTTGGCCACTCCTCTTCCT-3′, reverse 5′-GCCCTCTGTCCTTTTCCAAC-3′; *Tcf7*, forward 5′-GCTGGCTGGACCTCCCTATT-3′, reverse 5′-GGGCAGGAGAAGCATTTGTA-3′; *Lef1*, forward 5′-AAGGAACACTGACAG CAACC-3′, reverse 5′-AGTCGGCGCTTGCAGTAGAC-3′; *Jun*, forward 5′-TCTACGCCA ACCTCAGCAAC-3′, reverse 5′-CTGTTCCCTGAGCATGTTGG-3′; *Fzd7*, forward 5′-CTG CACCATCCTCTTCATGG-3′, reverse 5′-CTAGACAGGCCCACGTAGCA-3′; *LRP5*, forward 5′-GTGAGCCTCCTACCTGCTCC-3′, reverse 5′-ACATACTCGTGGGGAAAGG G-3′; *LRP6*, forward 5′-CCAATTGCTTTGGCTCTTGA-3′, reverse 5′-CGGGACAATTGA GTTCATCG-3′; and *GAPDH*, forward 5′-ACATGGCCTCCAAGGAGTAA-3′, reverse 5′-GGT CTGGGATGGAAATTGTG-3′.

### Statistical analysis

The mean location densities of gel bands were determined using Image J (NIH, Bethesda, MD, USA). The relative expression levels of AKT, p-AKT, p-ERK, p-GSK-3β, β-catenin, and FOXO1 were calculated by dividing the densitometry value of each protein by the value of β-actin. TCF1 signals were expressed as the relative intensities of each stimulatory vs. inhibitory TCF1. All data were analyzed with the statistical program, Prism 4.0 GraphPad (San Diego, CA). Student's t-test was used to determine the statistical significance of differences between groups.

## Results

### 4-1BB triggering induces GSK-3β/β-catenin signaling in activated CD8^+^ T cells with delayed kinetics

**Figure 1:**
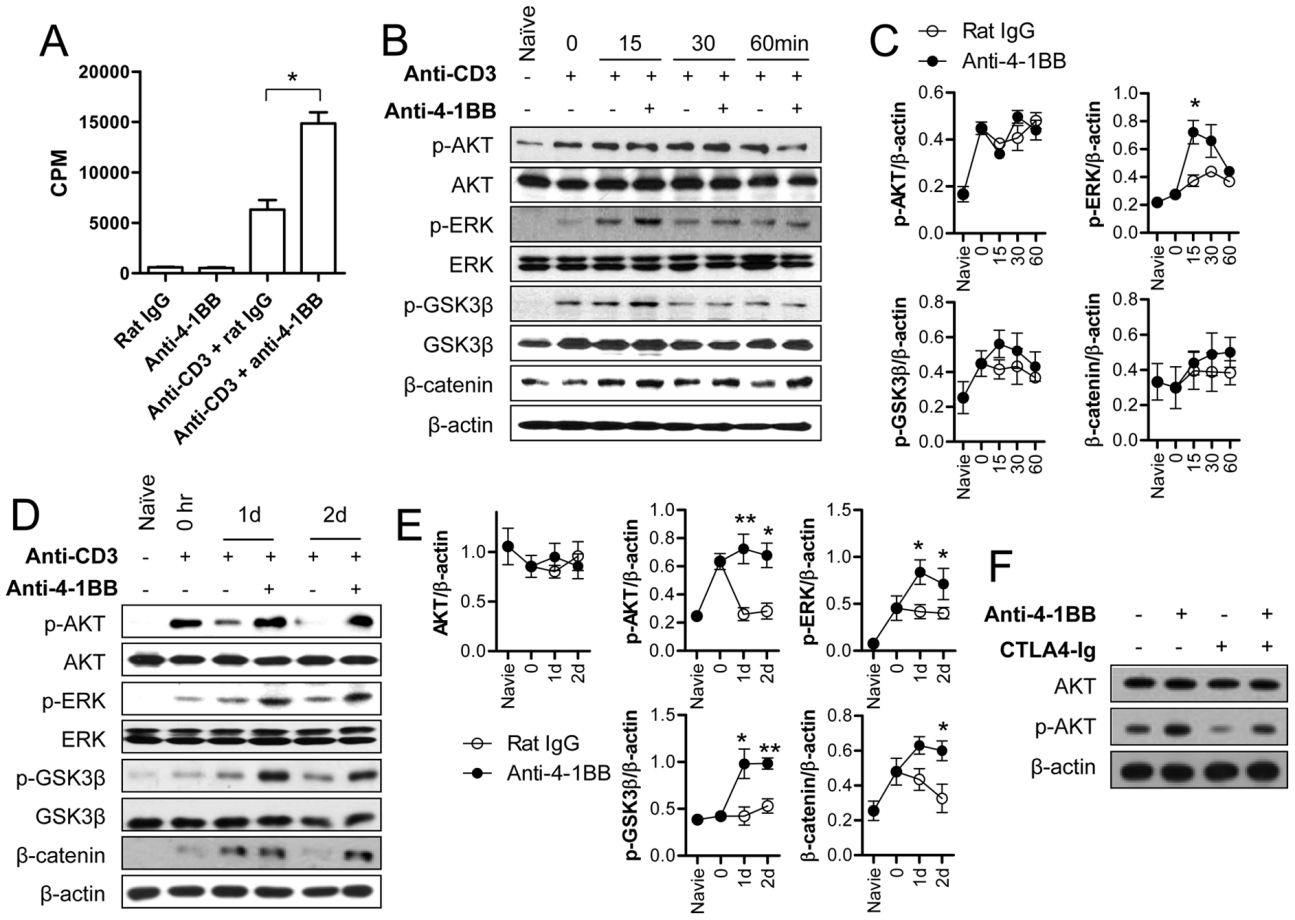
4-1BB sustains anti-CD3 induced signals to induce GSK-3/β-catenin signaling with delayed kinetics. The purified CD8^+^ T cells from C57BL/6 mice were activated with 0.1 µg/ml of anti-CD3 for 16 h. (A) CD8^+^ T cells cultured with or without anti-CD3 for 16 h were further incubated with 5 µg/ml of anti-4-1BB or rat IgG for another 48 h, and cell proliferation was assessed by [^3^H]-thymidine incorporation. (B–C) The activated CD8^+^ T cells were incubated with 5 µg/ml of anti-4-1BB or rat IgG for 0, 15, 30, or 60 min. Western blot analysis was performed with p-AKT, p-ERK, p-GSK-3β, β-catenin, and β-actin antibodies (B) and the relative expression levels of p-AKT, p-ERK, p-GSK-3β, and β-catenin were determined (C). (D–E) The activated CD8^+^ T cells were stimulated with 5 µg/ml of anti-4-1BB for 0, 1, or 2 days. Western blotting was performed (D) and the relative expression levels of each protein was determined (E). (F) The activated CD8^+^ T cells were treated with 5 µg/ml anti-4-1BB or rat IgG following 1 h incubation with 10 µg/ml CTLA-4Ig. After 24 h incubation, CD8^+^ T cells were harvested, lysed and subjected to Western blot analysis using AKT, p-AKT, and β-catenin antibodies. Naïve indicates CD8^+^ T cells that were cultured in complete medium for 16 h without anti-CD3. Results in C and E are mean ±SD of three independent experiments and representative data of two independent experiments are shown in F (*p<0.05; **p<0.01).

4-1BB signaling typically enhances the proliferation of CD8^+^ T cells through the PI3K/ERK pathway [3], [4], while GSK-3 negatively regulates the proliferation of antigen-specific CD8^+^ T cells [17]. Therefore, we first examined whether 4-1BB signaling would inactivate GSK-3β via the phosphorylation of the regulatory serine residue at position 9. CD8^+^ T cells from C57BL/6 mice were first stimulated with anti-CD3 for 16 h to induce 4-1BB on their surface and the 4-1BB-expressing CD8^+^ T cells were further incubated with agonistic anti-4-1BB. Under these conditions, the proliferation of activated CD8^+^ T cells was enhanced by 4-1BB triggering, as previous studies reported (Fig. 1A) [3], [4]. Lysates of the activated CD8^+^ T cells that were stimulated with anti-4-1BB or rat IgG for 0, 15, 30, or 60 min were subjected to Western blotting to assess the phosphorylation of AKT, ERK, and GSK-3β, as well as levels of β-catenin. 4-1BB triggering did not change the phosphorylation of AKT and GSK-3β as well as the level of β-catenin, but did increase the phosphorylation of ERK at the early time points (Fig. 1B and C). However, when the activated CD8^+^ T cells were stimulated with anti-4-1BB for 1 or 2 days, 4-1BB signaling blocked the decrease of AKT phosphorylation and increased the phosphorylation of ERK and GSK-3β, and the level of β-catenin (Fig. 1D and E). Since anti-TCR and anti-CD28 synergistically activate AKT signal of CD8^+^ T cells [20], we examined whether B7.2 upregulation of activated CD8^+^ T cells was responsible for the delayed AKT phosphorylation. Therefore, the activated CD8^+^ T cells were stimulated with anti-4-1BB in the presence or absence of CTLA-4Ig for 24 h. Western blot analysis indicated that 4-1BB signaling still enhanced AKT phosphorylation of CD8^+^ T cells in the absence of CD28–B7 signaling (Fig. 1F). These results indicate that 4-1BB triggering induced GSK-3β/β-catenin signaling in activated CD8^+^ T cells with delayed kinetics.

**Figure 2:**
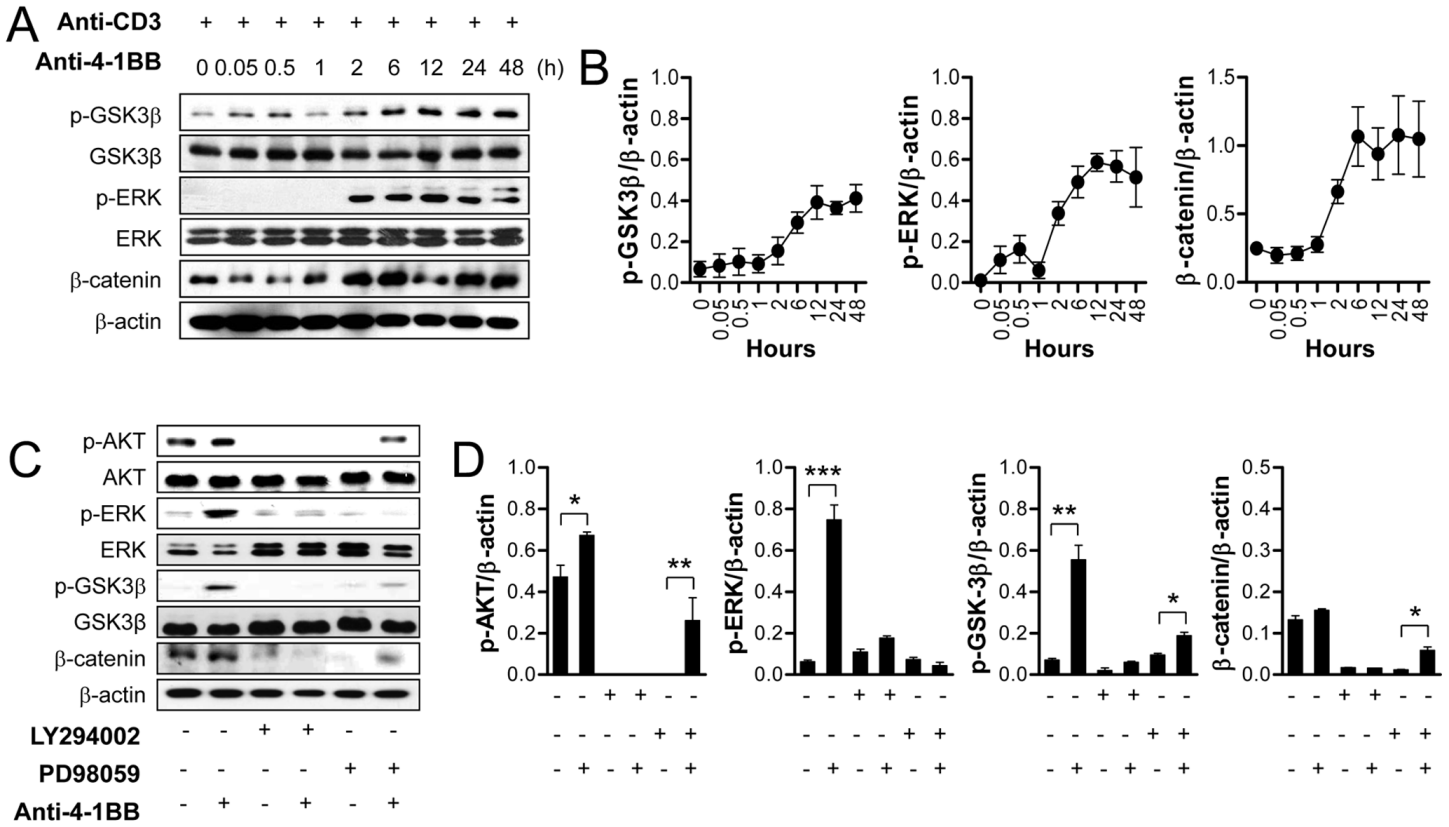
GSK-3/β-catenin signaling downstream of anti-CD3 plus anti-4-1BB is dependent on PI3K-ERK pathway. (A–B) CD8^+^ T cells activated by anti-CD3 for 16 h were stimulated with 5 µg/ml of anti-4-1BB for the indicated times. Western blotting was performed with p-GSK-3β, p-ERK, β-catenin, and β-actin antibodies (A), and the relative expression levels were calculated by dividing the densitometry value of each protein by the value of β-actin (B). (C–D) The activated CD8^+^ T cells were pre-incubated with or without 10 µM LY294002 or 10 µM PD98509 and then stimulated with 5 µg/ml of anti-4-1BB or rat IgG for 1 day. Western blotting was performed with p-AKT, p-GSK-3β, p-ERK, and β-catenin antibodies (C) and the relative expression levels of each protein was determined (D). Results in B and D are mean ±SD of three separate experiments (*p<0.05; **p<0.01; ***p<0.001).

To examine when 4-1BB triggering began to induce the phosphorylation of GSK-3β and maintain β-catenin levels in CD8^+^ T cells, we examined the kinetics of phosphorylation of GSK-3β and ERK, as well as β-catenin levels, after 4-1BB triggering of anti-CD3-treated CD8^+^ T cells. 4-1BB signaling led to phosphorylation of GSK-3β and ERK, and accumulation of β-catenin about 2–6 h after 4-1BB triggering, and this was sustained for 48 h (Fig. 2A and B). This result indicates that 4-1BB signaling indirectly induces the phosphorylation of ERK and GSK-3β, and sustains β-catenin levels. Next, to investigate whether 4-1BB signaling activated the PI3K/ERK pathway and the GSK-3β/β-catenin pathway separately, activated CD8^+^ T cells were incubated with anti-4-1BB for 24 h in the presence of the PI3K inhibitor LY294002 or the ERK inhibitor PD98059. Again, 4-1BB triggering of activated CD8^+^ T cells for 24 h significantly increased the phosphorylation of AKT, ERK, and GSK-3β. Inhibition of PI3K completely abolished the effects of anti-CD3 and/or anti-4-1BB by lowering the phosphorylation of AKT, ERK, and GSK-3β as well as β-catenin accumulation (Fig. 2C and D). While inhibition of ERK completely reversed the anti-CD3 effects on AKT, ERK, and β-catenin, but partially blocked the anti-4-1BB effects because 4-1BB triggering still moderately increased the phosphorylation of AKT and GSK-3β as well as β-catenin in the presence of ERK inhibitor (Fig. 2C and D).

Taken together, these results indicate that 4-1BB triggering induced the rapid and delayed ERK activations, while AKT, GSK-3β, and β-catenin were activated only in the delayed phase in PI3K- and ERK-dependent manner. We therefore suggest that 4-1BB triggering induces signaling pathways in a ‘two wave’ fashion: a rapid and transient activation (the ‘first wave’) and a delayed and sustained activation (the ‘second wave’).

### 4-1BB signaling induces Wnt genes and downstream transcription factors

**Figure 3:**
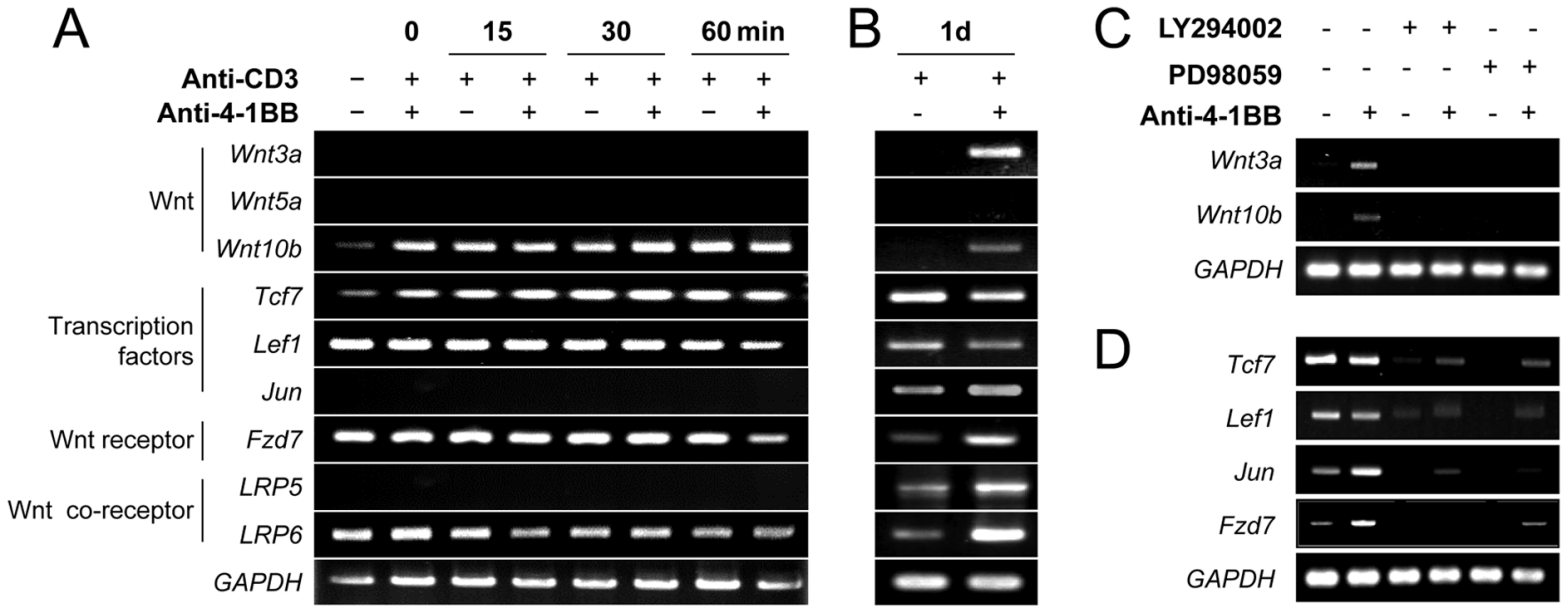
4-1BB triggering increases levels of Wnt ligands and their receptor. (A–B) CD8^+^ T lymphocytes were activated with anti-CD3 and then stimulated with anti-4-1BB or rat IgG for the indicated times. Total RNA was extracted from the CD8^+^ T cells and transcripts of *Wnts*, *Tcf7*, *Lef*, *Fzd*, *Jun*, and Wnt co-receptor LRP5/6 were measured by RT-PCR. *GAPDH* was used as an internal control. (C–D) The activated CD8^+^ T cells were pre-incubated with or without 10 µM LY294002 or 10 µM PD98059 for 1 h and then stimulated with 5 µg/ml anti-4-1BB or rat IgG for 24 h. Total RNAs were extracted and RT-PCR was performed with specific primer sets. Representative data from at least three independent experiments are shown.

The GSK-3β/β-catenin pathway is typically activated by Wnt proteins, and, of the various Wnt genes, *Wnt3a* and *Wnt10b* are involved in controlling T cell homeostasis [21], [22]. Since phosphorylation of GSK-3β was marginally increased by 4-1BB signaling after ERK blockade (Fig. 2C and D), it appears that ERK-independent signaling is partially involved in the 4-1BB-mediated GSK-3β phosphorylation. To clarify this issue, we measured the transcript levels of *Wnts*, the Wnt receptor and co-receptor, and Wnt signaling transcription factors such as *Lef* and *Tcf7* in CD8^+^ T cells that were stimulated *in vitro* with rat IgG or anti-4-1BB for 0, 15, 30, 60 min, or 24 h. Freshly isolated CD8^+^ T cells expressed *Wnt10b*, T cell factor 7 (*Tcf7*), lymphoid enhancer-binding factor-1 (*Lef1*), *Fzd7*, and *LRP6*, but not *Wnt3a*, *Wnt5a*, *Jun*, and *LRP5* (Fig. 3A). Anti-CD3 stimulation moderately increased *Wnt10b* and *Tcf7*, while 4-1BB stimulation showed transcript levels similar to the anti-CD3 level for the first 60 min (Fig. 3A). Anti-4-1BB, however, induced transcripts of *Wnt3a* and *Wnt10b* but not *Wnt5a* when they were measured for 24 h after the simulation (Fig. 3B). Transcript levels of *Tcf7* and *Lef1*, which are downstream transcription factors in the Wnt signaling pathway, were maintained in the activated CD8^+^ T cells independent of 4-1BB signaling (Fig. 3B). Transcript levels of *Jun*, of the Wnt receptor *Fzd7*, and of the Wnt co-receptor *LRP5/6* were increased in the activated CD8^+^ T cells by 4-1BB triggering (Fig. 3B).

To examine whether *Wnt3a* and *Wnt10b* were induced by 4-1BB signaling in the CD8^+^ T cells, activated CD8^+^ T cells were exposed to anti-4-1BB in the presence of LY294002 or PD98059. *Wnt3a* and *Wnt10b* transcripts failed to accumulate in the presence of the PI3K or ERK inhibitor (Fig. 3C). 4-1BB signaling did not enhance further *Tcf7*/*Lef1* transcription in these experiments. This could be because the expression of *Tcf7*/*Lef1* was already maximal in the activated CD8^+^ T cells (Fig. 3B); 4-1BB clearly induced some transcription of *Tcf7*, *Lef1*, and *Jun* in the presence of the PI3K or ERK inhibitor (Fig. 3D). On the other hand, 4-1BB-mediated increase of Fzd7 transcript was completely blocked by the PI3K inhibitor (Fig. 3D).

Taken together, these results suggest that 4-1BB signaling indirectly increases or maintains *Wnt3a* and *Wnt10b* expression as well as its receptor and coreceptor, and thus may support the activation of GSK-3β/β-catenin pathway in CD8^+^ T cells.

### 4-1BB signaling decreases FOXO1 and increases the stimulatory TCF1 with the delayed kinetics

**Figure 4:**
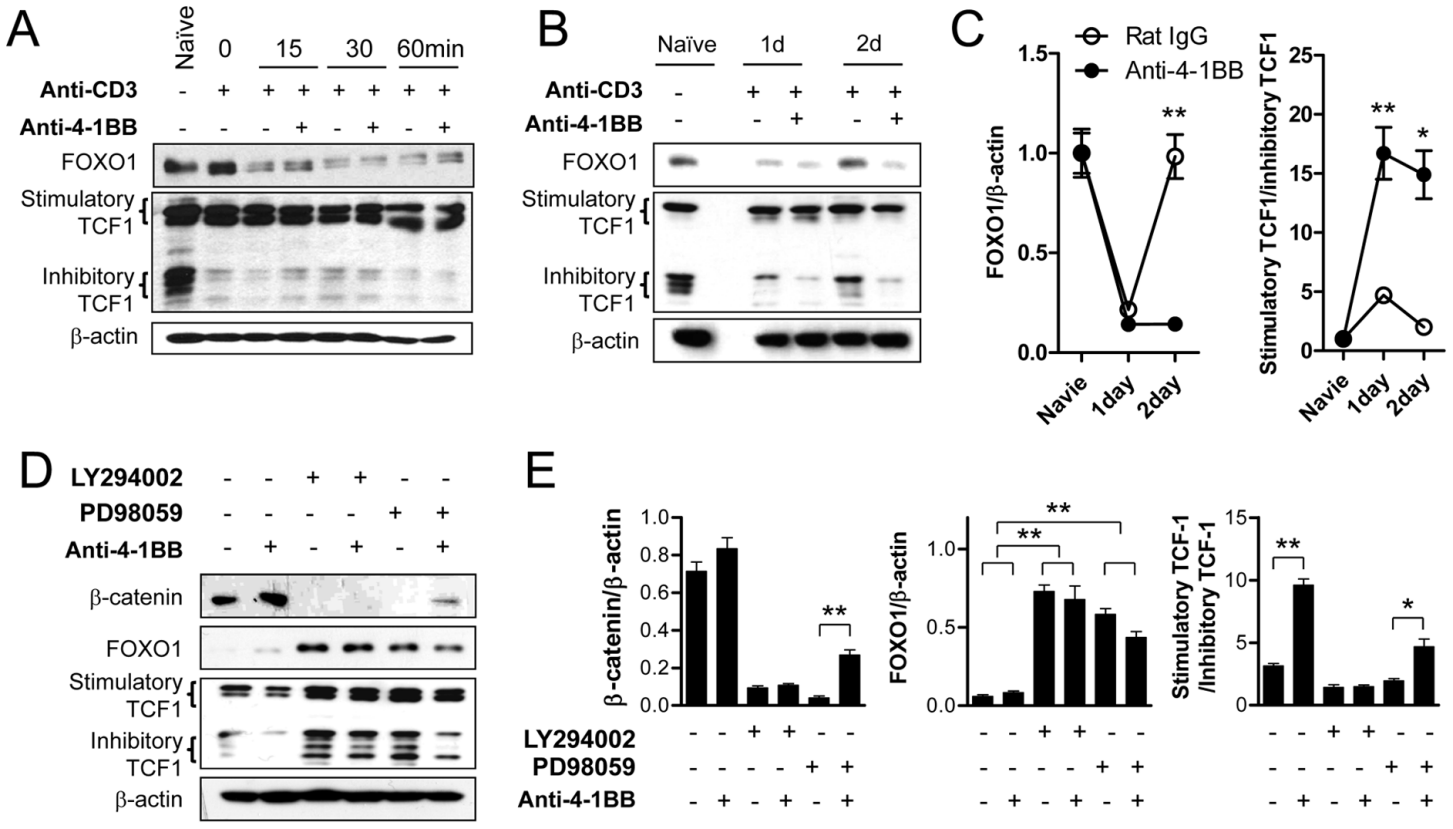
4-1BB signaling induces the delayed activation of β-catenin/TCF1 pathway under the control of PI3K-ERK pathway. (A–C) Anti-CD3-activated CD8^+^ T cells were stimulated with 5 µg/ml anti-4-1BB or rat IgG for the indicated times. Western blotting was performed with antibodies against β-catenin, TCF1, FOXO1 and β-actin. (A) Expression levels of FOXO1, TCF1 and β-catenin 0, 15, 30, or 60 min after 4-1BB triggering. (B) Expression levels of FOXO1, TCF1 and β-catenin 1 or 2 days after 4-1BB triggering. (C) The relative expression levels of FOXO1 and TCF1 on days 1 and 2 were calculated. (D–E) Activated CD8^+^ T cells were pre-incubated with or without 10 µM LY294002 or 10 µM PD98509 for 1 h and then stimulated with anti-4-1BB or rat IgG for 24 h. Western blotting was performed with β-catenin, FOXO1, TCF1, and β-actin antibodies (D), and the relative expression levels of β-catenin, FOXO1 and TCF1 were calculated (E). Naïve indicates CD8^+^ T cells that were cultured in complete medium for 16 h without anti-CD3. Results in C and E are mean ±SD of three separate experiments (*p<0.05; **p<0.01).

β-catenin plays a crucial role in many developmental processes by interacting with transcription factors such as TCF1, FOXO1 and SMAD [23], [24]. In the context of CD8^+^ T cell biology, FOXO1 is up-regulated in naïve CD8^+^ T cells and regulates the maintenance and life span of these cells by sensing growth factor availability [25]. Once CD8^+^ T cells are activated, TCF1 is required for primary expansion of mature CD8^+^ T cells and crucial for the formation of memory CD8^+^ T cells [26]. Therefore, we measured expression of FOXO1 and TCF1 in activated CD8^+^ T cells following 4-1BB triggering. As previously reported [25], [27], naïve CD8^+^ T cells maintained a high level of FOXO1, the stimulatory TCF1 isoform, and the inhibitory TCF1 ΔCTNNB isoform (Fig. 4A). Anti-CD3 alone gradually decreased FOXO1 and inhibitory TCF1 but not stimulatory TCF1. Additional stimulation with anti-4-1BB did not alter the anti-CD3-mediated levels of FOXO1 and TCF1 in the activated CD8^+^ T cells during the first 60 min (Fig. 4A). When the CD8^+^ T cells were exposed to anti-4-1BB or rat IgG for 1–2 days, the reduced FOXO1 and inhibitory TCF1 were recovered by day 2 in rat IgG− but not in anti-4-1BB-treated activated CD8^+^ T cells (Fig. 4B). Statistical analysis indicated that 4-1BB triggering significantly decreased FOXO1 level by day 2 and increased the ratio of stimulatory to inhibitory TCF1 by day 1 and 2 compared with that of rat IgG-treated CD8^+^ T cells (Fig. 4C). These results indicate that 4-1BB signaling blocks the recovery of FOXO1 and inhibitory TCF1 in the activated CD8^+^ T cells.

To examine the role of PI3K and ERK in the 4-1BB-mediated regulation of β-catenin, FOXO1, and TCF1, we preincubated anti-CD3-activated CD8^+^ T cells with the PI3K or ERK inhibitor and then incubated them with anti-4-1BB only for 24 h because 48 h of culture with inhibitors markedly reduced the number of viable cells. The effects of 4-1BB signaling on the ratio of stimulatory to inhibitor TCF1 were completely abolished by the PI3K inhibitor, and significantly reduced by the ERK inhibitor (Fig. 4D and E). In this experiment, 4-1BB did not show significant effects on FOXO1 or β-catenin over and above the effects of anti-CD3 alone because the effects of anti-4-1BB became significant by day 2 rather than day 1 (Fig. 4B). However, the anti-CD3-induced changes were also ERK and PI3K dependent even in the presence of anti-4-1BB mAb.

These results suggest that 4-1BB signaling maintains the stimulatory TCF1 and β-catenin at high levels, and FOXO1 and inhibitory TCF1 at low levels in CD8^+^ T cells. Like AKT and GSK-3β, 4-1BB triggering only modulated the FOXO1 and TCF1 levels during the ‘second wave’ mainly through the PI3K/ERK pathway.

### 4-1BB is unique among TNFRSF members in increasing β-catenin and the ratio of stimulatory to inhibitory TCF1 in CD8^+^ T cells

**Figure 5:**
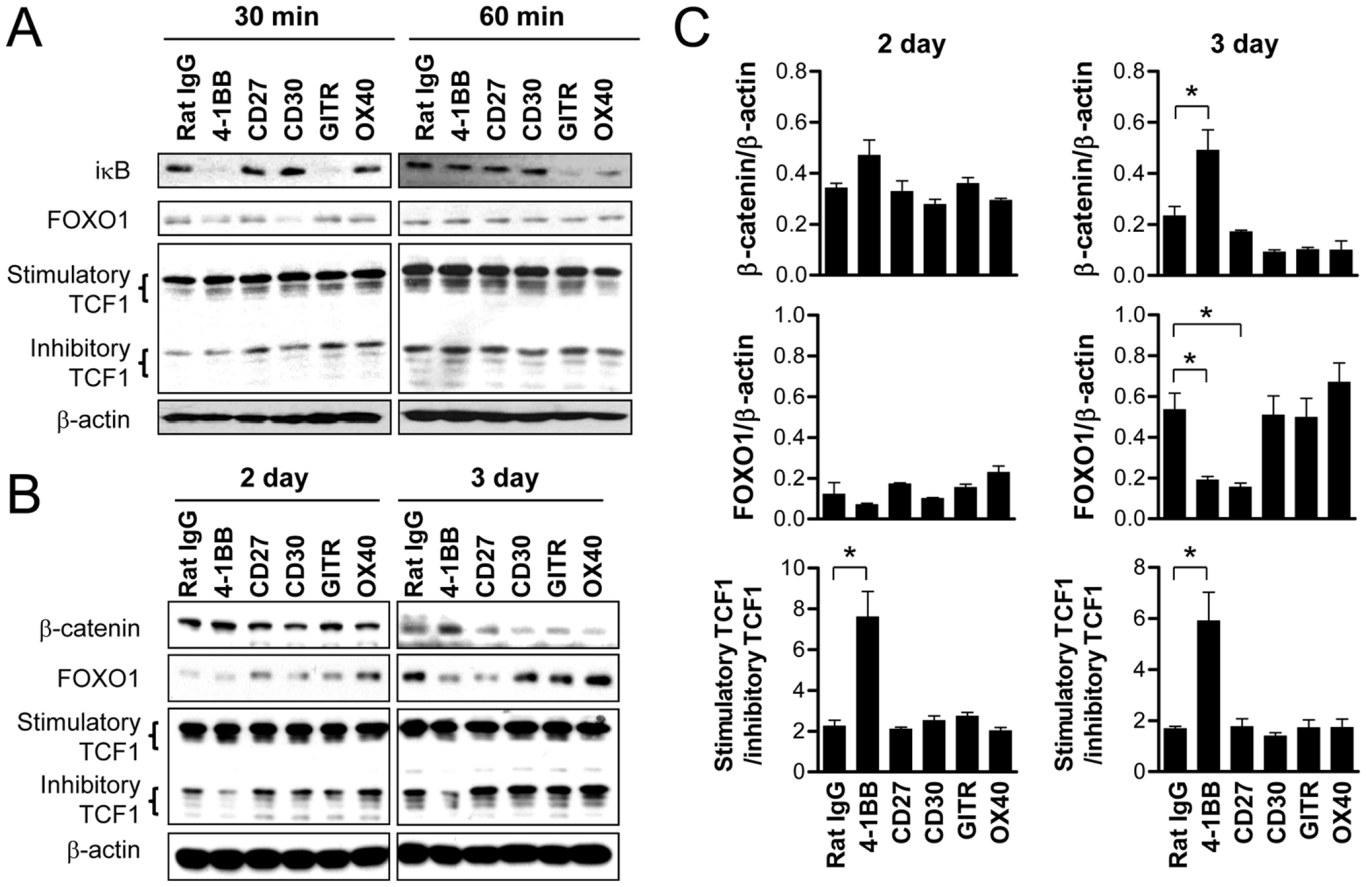
4-1BB is unique among TNFRSF members in activating the β-catenin/TCF1 pathway in CD8^+^ T cells. (A) CD8^+^ T cells activated by anti-CD3 for 16 h were stimulated with 5 µg/ml of anti-4-1BB, anti-CD27, and anti-CD30, or 10 µg/ml anti-GITR and anti-OX40 for 30 or 60 min. Western blot analysis was performed using IκB, FOXO1, TCF1, and β-actin antibodies. (B–C) Freshly isolated CD8^+^ T cells were activated with 0.1 µg/ml of anti-CD3 and simultaneously treated with 5 µg/ml of anti-4-1BB, anti-CD27, and anti-CD30, or 10 µg/ml anti-GITR and anti-OX40 for 2 or 3 days. Western blotting was performed with antibodies specific for β-catenin, TCF1 and FOXO1 and β-actin (B). The relative expression levels of β-catenin, FOXO1 and TCF1 were calculated (C). Results are mean ±SD of two separate experiments (*p<0.05).

We assessed whether 4-1BB signaling was unique among TNFRSF members in its ability to regulate the level of β-catenin, FOXO1, and the ratio of stimulatory to inhibitory TCF1. We compared this regulation ability of 4-1BB with other TNFRSF members such as CD27, CD30, GITR, and OX40. We first stimulated CD3-activated CD8^+^ T cells with agonistic antibodies to CD27, CD30, GITR or OX40 for 30 and 60 min to examine their direct effects on FOXO1 and TCF1 expression along with IκB degradation. Signaling through 4-1BB, GITR, and OX40 induced IκB degradation in activated CD8^+^ T cells within 30 or 60 min, while CD27 and CD30 triggering was not effective (Fig. 5A). Murine CD27 is constitutively expressed by naïve CD8^+^ T cells, plays a role in the priming of T cells, and uses both canonical and non-canonical NF-κB signaling pathways [12], [28]–[29]. The induction of CD30 is known to require CD28 and cytokine signaling, and peaks 3–4 days after T cell activation [30], [31]. Therefore, it appears that CD27 triggering activated non-canonical NF-κB signaling pathway rather than canonical pathway and anti-CD3 activation for 16 h was not enough to fully induce CD30 on CD8^+^ T cells to induce IκB degradation.

Because each member of TNFRSF shows different expression patterns, we activated freshly purified CD8^+^ T cells with anti-CD3, incubated them with antibodies against each member of TNFRSF for 2–3 days, and assessed the expressions of β-catenin, FOXO1, and TCF1. Although CD27 triggering significantly decreased FOXO1 expression by day 3 (Fig. 5C) [25], the rest of TNFRSF members except 4-1BB were largely ineffective in altering the expressions of β-catenin and FOXO1, and the ratio of stimulatory to inhibitory TCF1 (Fig. 5B and C). These results indicate that 4-1BB is unique in maintaining the stimulatory TCF1 and β-catenin at high levels in CD8^+^ T cells.

### 4-1BB signaling regulates the levels of β-catenin, FOXO1, and TCF1 in CD8^+^ T cells but not in CD4^+^ T cells

**Figure 6:**
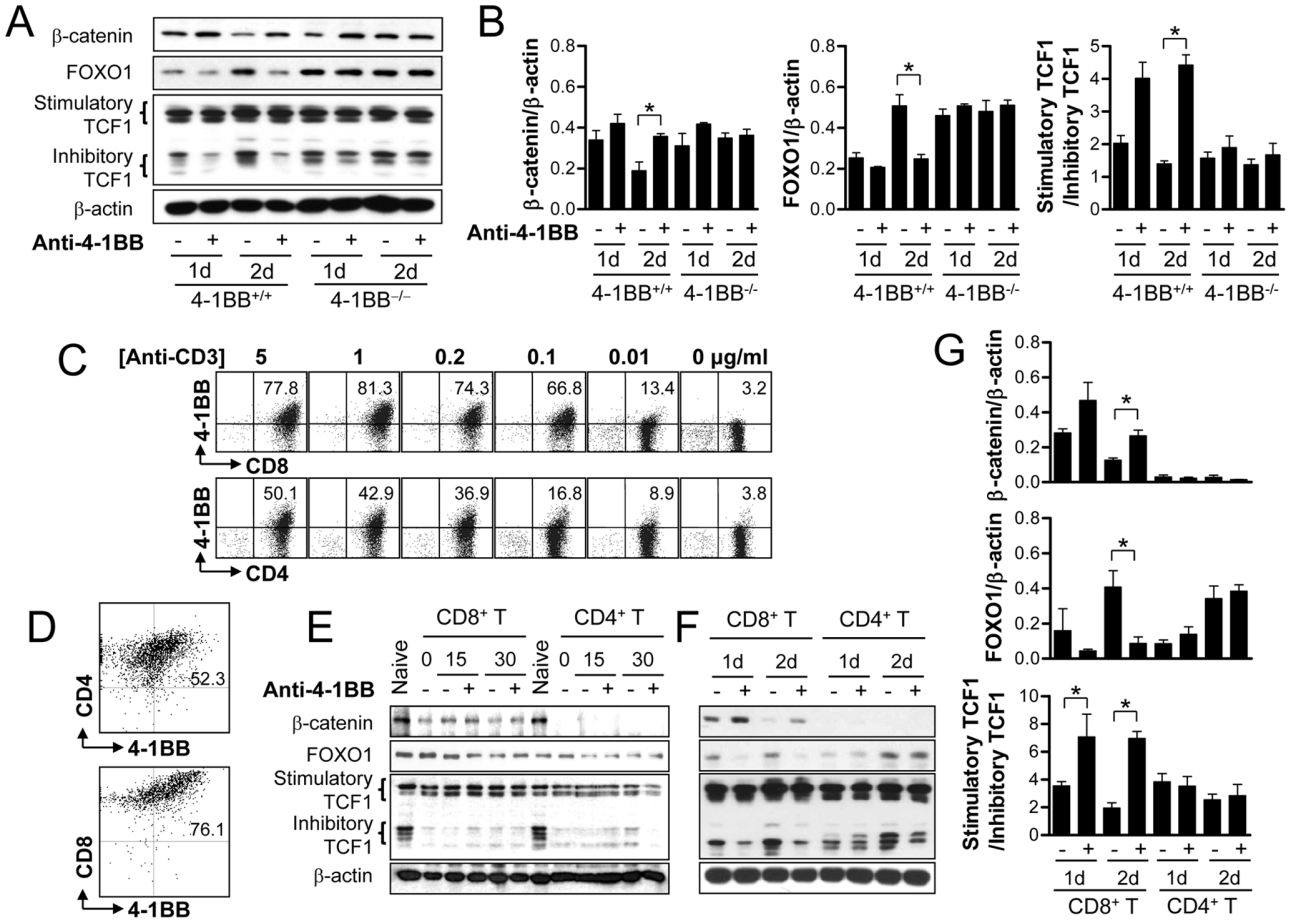
4-1BB signaling activates the β-catenin/TCF1 pathway in CD8^+^ T cells but not in CD4^+^ T cells. (A–B) CD8^+^ T cells were isolated from WT and 4-1BB^−/−^ C57BL/6 mice, activated with anti-CD3 for 16 h, and incubated with anti-4-1BB for 1 or 2 days. Western blotting was performed with antibodies specific for β-catenin, TCF1, FOXO1 and β-actin (A). The relative expression levels of β-catenin, FOXO1 and TCF1 were calculated (B). (C) Purified CD4^+^ and CD8^+^ T cells were activated with the indicated dose of anti-CD3 mAb for 24 h. The activated T cells were stained with FITC-conjugated anti-CD4 or anti-CD8 mAb along with PE-conjugated anti-4-1BB mAb (eBioscience) and subsequently analyzed by FACSCalibur (BD Bioscience). (D–G) CD8^+^ and CD4^+^ T cells were activated for 16 h and 36 h, respectively in the presence of 0.1 µg/ml anti-CD3, and then stimulated with 5 µg/ml 4-1BB for the indicated times. (D) FACS analysis of CD4^+^ and CD8^+^ T cells. (E) Expression levels of FOXO1, TCF1 and β-catenin in activated CD8^+^ T and CD4^+^ T cells 0, 15, or 30 min after 4-1BB triggering. (F) Expression levels of FOXO1, TCF1 and β-catenin in activated CD8^+^ T and CD4^+^ T cells 1 or 2 days after 4-1BB triggering. (G) The relative expression levels of β-catenin, FOXO1 and TCF1 were calculated from E. Naïve indicates CD8^+^ T cells that were cultured in complete medium for 16 h without anti-CD3. Results are mean ±SD of three separate experiments (*p<0.05).

4-1BB typically functions as a potent costimulatory molecule enhancing CD8^+^ T cell responses *in vitro* and *in vivo* [1], [2], and modulates the levels of β-catenin, FOXO1, and TCF1. Thus we next investigated the expression patterns of β-catenin, TCF1, and FOXO1 in CD8^+^ T cells from 4-1BB^−/−^ mice. CD8^+^ T cells from wild-type and 4-1BB^−/−^ C57BL/6 mice were activated with anti-CD3 to induce the 4-1BB expression for 16 h and further stimulated with anti-4-1BB for 1 or 2 days. As shown before, 4-1BB stimulation of the 4-1BB-sufficient CD8^+^ T cells sustained the β-catenin level and led to a continuous decline in FOXO1 and inhibitory TCF1 (Fig. 6A). Surprisingly, however, the expressions of β-catenin, FOXO1 and inhibitory TCF1 were not decreased at all in the 4-1BB^−/−^ CD8^+^ T cells following activation with anti-CD3, and the basal levels of FOXO1 and inhibitory TCF1 were higher in 4-1BB^−/−^ CD8^+^ T cells compared with those of 4-1B-intact CD8^+^ T cells (Fig. 6A).

Although CD4^+^ T and CD8^+^ T cells both express 4-1BB to similar extents on their surfaces, 4-1BB signaling is less effective in enhancing the proliferation of CD4^+^ T cells than that of CD8^+^ T cells *in vitro* and *in vivo* [32], [33]. Therefore, we next examined whether CD4^+^ T cells modulate the levels of β-catenin, FOXO1, and TCF1 in response to 4-1BB signaling. Anti-CD3 activation was less effective in inducing 4-1BB on CD4^+^ T cells than on CD8^+^ T cells (Fig. 6C). Activation of T cells with 0.1 µg/ml of anti-CD3 for 16 h routinely resulted in ∼70% of 4-1BB expression on CD8^+^ T cells but only 10–20% on CD4^+^ T cells. If anti-4-1BB was added to the activated CD4^+^ T cells that included 10–20% of 4-1BB^+^ cells, it would not be clear whether 4-1BB triggering was indeed ineffective in modulation of FOXO1, β-catenin, and TCF1 of CD4^+^ T cells. Therefore, CD4^+^ T cells were cultured with anti-CD3 for 36 h to induce 4-1BB on >50% cells (Fig. 6D) and further stimulated with anti-4-1BB. 4-1BB triggering for 15 or 30 min did not alter the levels of FOXO1, β-catenin, and TCF1 in both CD8^+^ T and CD4^+^ T cells (Fig. 6E). 4-1BB triggering for 1 or 2 days again blocked the decrease of β-catenin and suppressed the recovery of FOXO1 and inhibitory TCF1 in the activated CD8^+^ T cells but these 4-1BB effects were not observed at all in the activated CD4^+^ T cells (Fig. 6F). Surprisingly the activated CD4^+^ T cells barely expressed β-catenin under any conditions, and 4-1BB signaling failed to reduce FOXO1 and inhibitory TCF1 in CD4^+^ T cells. As in the CD8^+^ T cells, FOXO1 and inhibitory TCF1 transiently decreased in the activated CD4^+^ T cells, but their levels fully recovered by day 2, and 4-1BB signaling did not block this recovery (Fig. 6G).

These results indicate that 4-1BB signaling only modulates the levels of β-catenin, FOXO1, and TCF1 of CD8^+^ T cells, but not of CD4^+^ T cells.

### 4-1BB signaling enhances the proliferation and IFN-γ production of CD8^+^ T cells through β-catenin/TCF1 interaction

**Figure 7:**
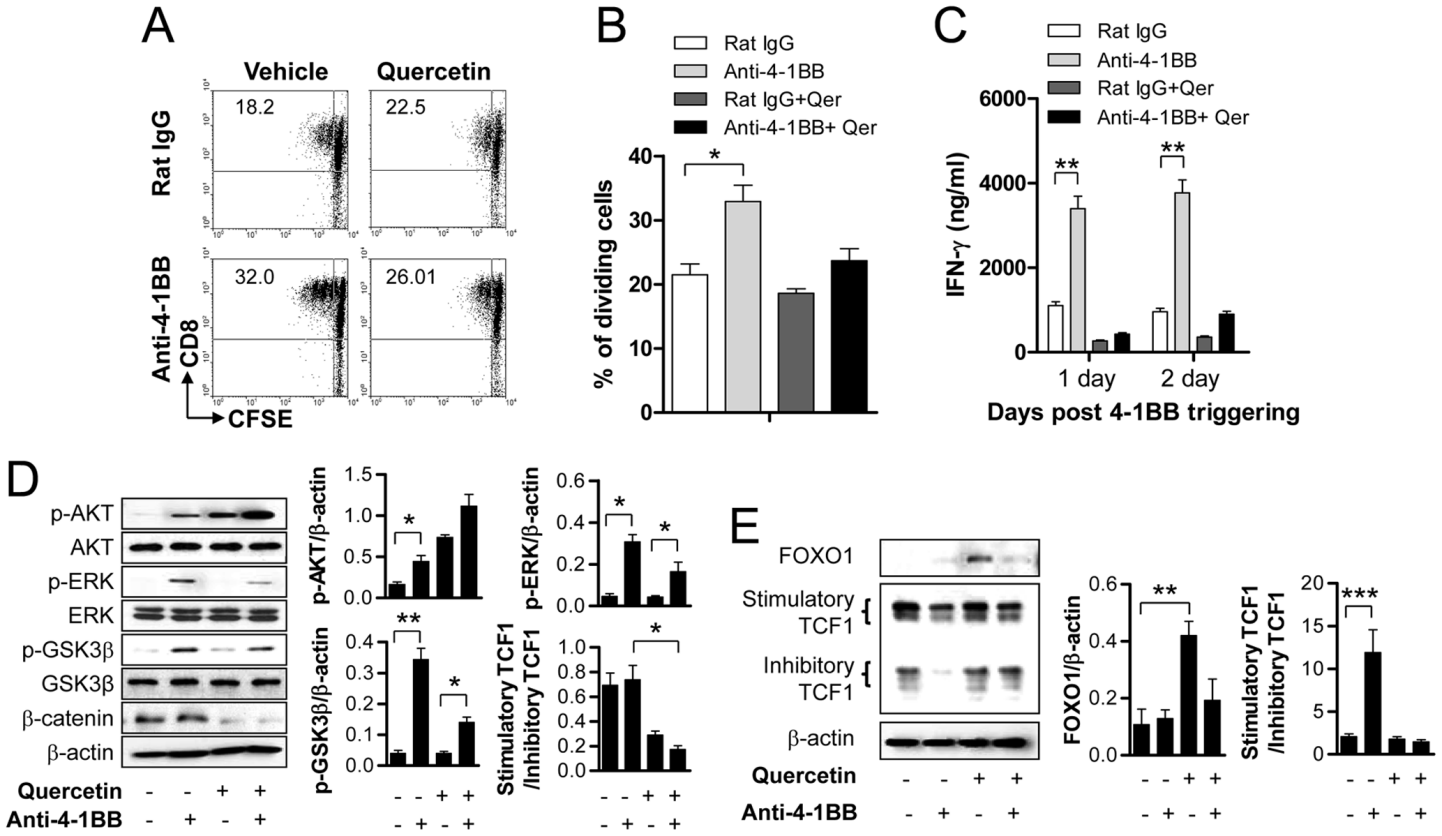
4-1BB signaling enhances the proliferation of CD8^+^ T cells and their IFN-γ production through β-catenin/TCF1 interaction. (A–B) Purified CD8^+^ T cells labeled with CFSE were incubated with 0.1 µg/ml of anti-CD3 for 16 h. The activated CD8^+^ T cells were pre-incubated with or without 25 µM quercetin for 1 h and then stimulated with anti-4-1BB or rat IgG for 48 h. Cell proliferation was assessed by the CFSE dilution method. Live cells were gated, and plotted as CFSE vs. CD8 (A), and proportions of the dividing cells were determined (n = 3) (B). (C) Activated CD8^+^ T cells were pre-incubated with or without 25 µM quercetin for 1 h and then stimulated with anti-4-1BB or rat IgG for 24 h or 48 h. The IFN-γ concentration in cell supernatants was determined using a BD™ Cytometric Bead Array kit (BD Bioscience). (D, E) Activated CD8^+^ T cells were pre-incubated with or without 25 µM quercetin for 1 h and then stimulated with anti-4-1BB or rat IgG for 24 h. Western blotting was performed with specific antibodies and the relative expression levels of p-AKT, p-ERK, p-GSK-3β and β-catenin were calculated. Results are mean ±SD of three separate experiments (*p<0.05; **p<0.01).

Quercetin is a plant-derived flavonoid found in fruits, vegetables, leaves and grains, and an inhibitor of β-catenin/TCF1 transcriptional activity [34]. To examine whether 4-1BB signaling enhances the proliferation of CD8^+^ T cells through the β-catenin/TCF1 pathway, CFSE-labeled and anti-CD3-activated CD8^+^ T cells were pre-incubated with quercetin and then stimulated with anti-4-1BB. The result showed that 4-1BB signaling increased the number of dividing cells compared with that of rat IgG-treated CD8^+^ T cells (Fig. 7A). The treatment of quercetin clearly decreased the number of dividing cells in anti-4-1BB-treated CD8^+^ T cells, but not rat IgG-treated CD8^+^ T cells (Fig. 7A and B). Moreover, quercertin completely blocked 4-1BB-mediated enhancement of IFN-γ production from CD8^+^ T cells (Fig. 7C).

We further examined the phosphorylation of signaling molecules involved in the 4-1BB-mediated signaling pathway. Treatment of quercertin enhanced the phosphorylation of AKT in both rat IgG− and anti-4-1BB-treated CD8^+^ T cells or moderately blocked the 4-1BB-mediated increase of ERK and GSK-3β phosphorylation, while this quercetin treatment completely abolished the level of β-catenin (Fig. 7D). In case of FOXO1 and TCF1, quercetin did not reverse the 4-1BB-mediated decrease of FOXO1, rather increased FOXO1 expression in rat IgG-treated CD8^+^ T cells, and selectively abolished the decrease of inhibitory TCF1 by 4-1BB signaling (Fig. 7E). Quercetin abolished the 4-1BB-mediated increase of β-catenin and decrease of inhibitory TCF1 in CD8^+^ T cells, but did not change the modulation of GSK-3β phosphorylation and FOXO1 decrease by anti-4-1BB. These results were expected because we suspected that a molecule induced by 4-1BB signaling would modulate the levels of β-catenin, FOXO1, and TCF1 through the PI3K/AKT/ERK pathway. However, it is not clear why AKT and ERK phosphorylation, the upstream molecules in the 4-1BB-meidated GSK-3β phosphorylation, were increased or decreased by quercertin. Taken together, these results suggest that the delayed activation of the β-catenin/TCF1 pathway by 4-1BB triggering is involved in the enhanced proliferation of CD8^+^ T cells.

## Discussion

We found that 4-1BB signaling first induced rapid activation of ERK signals by amplifying anti-CD3-activated mitogenic signals as the ‘first wave’ (Fig. 1B) [35], and this led to the delayed activation of PI3K/AKT/ERK signals as the ‘second wave’ signal and eventually enhanced the proliferation of CD8^+^ T cells by inactivating GSK3β and inducing β-catenin/TCF1 signals (Fig. 1, 2, and 4) [18]. Indeed, quercetin, a potent inhibitor of β-catenin/TCF1 signaling, completely abolished the 4-1BB effect on CD8^+^ T cell proliferation (Fig. 7). 4-1BB was the most effective among the TNFRSF members in activating the β-catenin/TCF1 pathway only in CD8^+^ T cells but not in CD4^+^ T cells (Fig. 5 and 6). These results elucidate how 4-1BB signaling enhances the proliferation of activated CD8^+^ T cells and this may promote the differentiation of CD8^+^ T cells into effector or memory cells.

As previous studies suggested [36], [37], signaling through the TCR/CD3 complex only transiently activated mitogenic signaling in the CD8^+^ T cells and further signaling via costimulatory molecules was required to amplify and sustain the mitogenic signals needed for continuous proliferation and differentiation of the CD8^+^ T cells. TCR/CD3 signaling not only transiently generated mitogenic signals; it also transiently triggered the GSK-3β/β-catenin/TCF1 signal axis leading to proliferation of CD8^+^ T cells (Fig. 1B, 1D, 4A, 4B, and 7). 4-1BB signaling intensified the GSK-3β/β-catenin signals with the delayed kinetics, which was mainly dependent on the PI3K/ERK pathway and also to some extent on the ERK-independent pathway (Fig. 2C and D). Moreover, 4-1BB signaling selectively induced the expression of *Wnt3a*, and sustained *Wnt10b* and their receptor *Fzd7* through PI3K/ERK signal-dependent and -independent pathways (Fig. 3). Therefore, 4-1BB signaling seems to induce the delayed activation of GSK-3β/β-catenin signals that enhance CD8^+^ T cell proliferation by three routes: reactivation of TCR/CD3-mediated signaling, ERK-independent activation of GSK-3β/β-catenin signaling, and induction of Wnt ligands. Among these three routes, 4-1BB signaling mainly used the reactivation of TCR/CD3-mediated signals to activate GSK-3β/β-catenin signals. Since we assessed the phosphorylation of GSK-3β and the expression of β-catenin, FOXO1 and TCF1 by Western blot analysis, it is not clear whether 4-1BB signaling regulates the expression of these proteins in all CD8^+^ T cells or affects only a specific subpopulation of CD8^+^ T cells.

β-catenin plays a dual role in regulating Wnt and NF-κB signaling pathways by activating Wnt target gene expression and inhibiting NF-κB-mediated transcriptional activation [38], [39]. Therefore, the sustained levels of β-catenin by 4-1BB signaling may not only promote the proliferation of CD8^+^ T cells along with the increase of stimulatory TCF1, but also potentially reduce the survival rate of activated CD8^+^ T cells by inhibiting NF-κB activation. Given that 4-1BB signaling is currently employed to treat cancers in the clinic in the forms of agonistic anti-4-1BB or chimeric antigen receptors containing the 4-1BB cytoplasmic domain to enhance anti-tumor CD8^+^ T cell responses [5], [40], the cross-talk between the Wnt and NF-κB signaling pathways upon 4-1BB stimulation needs to be fully elucidated to understand the therapeutic mechanisms of 4-1BB.

Although CD4^+^ and CD8^+^ T cells express comparable levels of 4-1BB on their surfaces, 4-1BB signaling is less effective in CD4^+^ T cells than in CD8^+^ T cells [33]. Under the same *in vitro* conditions, CD4^+^ T cells require stronger TCR/CD3-mediated signals to express 4-1BB than CD8^+^ T cells (Fig. 6C). Although TCR/CD3 signaling caused transient degradation of FOXO1 and inhibitory TCF1 in CD4^+^ T cells, 4-1BB signaling did not affect the expression of FOXO1 and TCF1 in CD4^+^ T cells (Fig. 6E). Moreover, because CD4^+^ T cells barely express β-catenin even after 4-1BB triggering (Fig. 6E), it seems that β-catenin/TCF1 complexes minimally contribute to CD4^+^ T cell responses.

Each of the TNFRSF members such as 4-1BB, CD27, CD30, GITR and OX40 plays its own roles in T cell survival, proliferation, and memory formation [12]. For example, CD27 ligand enhances the proliferation and memory formation of CD4^+^ and CD8^+^ T cells, and CD27-mediated effects take place relatively early in the immune response [12]. OX40 is expressed on activated CD4^+^ rather than on CD8^+^ T cells and plays a more prominent role in CD4^+^ T cells [12]. GITR plays a crucial role in the regulation of Treg cells and functions as an adjuvant for conventional CD4^+^ and CD8^+^ T cells [12]. CD30 is predominantly expressed on activated CD4^+^ T cells and primarily modulates Th2 responses [12]. It seems that to induce optimal T cell responses to a given pathogen, expression of the different TNFRSF members needs to be spatially and temporally fine-tuned. 4-1BB is known to be a potent stimulator of activated CD8^+^ T cells, and 4-1BB signaling is more effective in triggering signaling in CD8^+^ T cells than in CD4^+^ T cells [33]. We currently suspect that 4-1BB signals preferentially induced CD8^+^ T cell responses by selectively activating the β-catenin/TCF1 pathway only in CD8^+^ T cells.

Stittrich et al. [41] demonstrated that miR-182 is induced by IL-2 in the late phase of CD4^+^ T cell expansion, targets the 3′ UTR of the mRNA for FOXO1, and thus decreases FOXO1 expression, the process leading to the clonal expansion of CD4^+^ T cells. This study implies that FOXO1, a well-known suppressor of clonal expansion, needs to be decreased in the late phase of T cell expansion for the clonal expansion. Therefore, we currently suspect that IL-2 would be the intervening molecule that induces β-catenin/TCF1 signaling in CD8^+^ T cells with delayed kinetics. Indeed, IL-2 production of CD8^+^ T cells was decreased in the absence of endogenous 4-1BB signaling and increased following 4-1BB triggering (data not shown). In addition, since TCF1 is required for the proliferation of mature CD8^+^ T cells and their differentiation into a central memory phenotype [26], we suspect that the delayed activation of the β-catenin/TCF1 pathway by 4-1BB signaling may be involved in the proliferation, differentiation into effector cells, and memory formation of CD8^+^ T cells *in vivo*, which is under our investigation.
